# PRADER-WILLI SYNDROME: WHAT IS THE GENERAL PEDIATRICIAN SUPPOSED TO DO? - A REVIEW

**DOI:** 10.1590/1984-0462/;2018;36;3;00003

**Published:** 2018

**Authors:** Caroline Buff Gouveia Passone, Paula Lage Pasqualucci, Ruth Rocha Franco, Simone Sakura Ito, Larissa Baldini Farjalla Mattar, Celia Priszkulnik Koiffmann, Leticia Azevedo Soster, Jorge David Aivazoglou Carneiro, Hamilton Cabral, Durval Damiani

**Affiliations:** a Instituto da Criança, São Paulo, SP, Brasil.; b Instituto de Ciências Biomédicas, São Paulo, SP, Brasil.; c Instituto Central do Hospital das Clinicas, São Paulo, SP, Brasil.

**Keywords:** Prader-Willi Syndrome, Growth hormone, Behavior, Diet, Treatment, Síndrome de Prader-Willi, Hormônio de crescimento, Comportamento, Dieta, Tratamento

## Abstract

**Objective:**

To carry out a review about Prader-Willi Syndrome based on the most recent data about the subject and to give recommendation for the general pediatricians for early diagnoses and follow-up.

**Data sources:**

Scientific articles in the PubMed and SciELO databases. The research was not limited to a specific time period and included all articles in such databases.

**Data synthesis:**

The Prader-Willi Syndrome (PWS) is a rare genetic disorder resulting from the loss of imprinted gene expression within the paternal chromosome 15q11-q13. PWS is characterized by endocrine abnormalities, such as growth hormone (GH) deficiency, obesity, central adrenal insufficiency, hypothyroidism, hypogonadism and complex behavioral and intellectual difficulties. PWS individuals also may present other comorbidities, such as sleep disorders, scoliosis, constipation, dental issues and coagulation disorders. The follow-up protocol of the Children’s Institute at Universidade de São Paulo is based on four main pillars: diet, exercise, recombinant human growth hormone (rhGH) therapy and behavioral and cognitive issues. The diet must include a caloric restriction of 900 kcal/day, according to the Prader-Willi Eating Pyramid and exercise plan is focused on daily aerobic exercises and postural therapy. The rhGH therapy is highly recommended by the international scientific literature and must be started as soon as the diagnostic is made. The management of behavioral issues is based on strategies to establish routine and rules.

**Conclusions:**

If the general pediatrician becomes more familiar with PWS, the diagnosis and treatment will start earlier, which is essential to improve the quality of life and care for these individuals.

## RESUMO

**Objetivo:**

Realizar uma revisão sobre a Síndrome de Prader-Willi (SPW) com base nas publicações mais recentes e fornecer recomendações ao pediatra geral para diagnóstico precoce e seguimento.

**Fonte de dados:**

Artigos publicados nas bases Pubmed e SciELO. A pesquisa não foi limitada a um período e incluiu todos os artigos das bases de dados.

**Síntese dos dados:**

A SPW é uma síndrome genética rara, resultante da perda do imprinting gênico expresso no cromossomo paterno 15q11-q13, sendo caracterizada por alterações endocrinológicas, como deficiência de hormônio de crescimento, obesidade, insuficiência adrenal central, hipotireoidismo, hipogonadismo, além de alterações comportamentais e déficit intelectual. Há outras comorbidades associadas, como distúrbios de sono, escoliose, constipação, problemas dentários e alterações de coagulação. O protocolo de seguimento da SPW do Instituto da Criança da Universidade de São Paulo se baseia em quarto pilares principais: dieta, exercício físico, terapia com hormônio de crescimento humano recombinante (rhGH) e manejo comportamental e cognitivo. A dieta deve ser restrita a 900 kcal/dia, de acordo com a Pirâmide Alimentar do Prader-Willi, e o exercício físico deve ser diário, aeróbico e postural. A terapia com rhGH é fortemente recomendada pela literatura científica internacional e deve ser iniciada assim que for realizado o diagnóstico da síndrome. O manejo do comportamento é realizado com estratégias para estabelecer rotina e regras.

**Conclusões:**

Se a SPW se tornar mais familiar ao pediatra geral, o diagnóstico e o tratamento começarão mais precocemente, o que irá melhorar a qualidade de vida e os cuidados desses pacientes.

## INTRODUCTION

The Prader-Willi Syndrome (PWS) is a rare genetic disorder resulting from the loss of gene expression within the paternal chromosome 15q11-q13. PWS has a prevalence rate of 1/10-30,000 and is characterized by endocrine abnormalities due to hypothalamic-pituitary insufficiency and complex physical, behavioral and intellectual difficulties.^1^

Nowadays, most developing countries can reach an early diagnosis, at around 8.6 weeks of life,^2^ however, even in centers of mean reference of the diagnosis, it happens at 3.9 years of age. The purpose of clinical diagnostic criteria has changed in the last decades.^3^ In 1993, when genetic tests were still very limited, a consensus was established that served as a guideline for the diagnosis and was known as the criteria of HOLM.^4^ Gunay-Aaygun et al., in 2001, aiming at an early diagnosis, proposed sufficient features of PWS, which should prompt DNA testing: hypotonia with poor sucking under 2 years of age, hypotonia with history of poor sucking and global developmental delay between 2-6 years of age, and hyperphagia and cognitive impairment after the age of 6 years.^3^ As the disease aspects may be non-specific or only appear over time, clinical criteria may often fail to identify PWS cases. However, in the last decades, molecular genetic tests became available for the definitive diagnosis of PWS, and clinical criteria were recognized as an initial screening for the indication of the genetic test.^3^

Three different genetic mechanisms can lead to PWS: paternal deletion within chromosome region 15q11-q13, which occurs in approximately 70% of the cases. Maternal uniparental disomy of chromosome 15 (UPD15), occurring in approximately 25% of the affected individuals, whereas about 2% of PWS patients have biparental inheritance of chromosome 15, but show abnormal methylation pattern and gene expression. These patients have a defect in the imprinting center. The recurrence risk in deletion and UPD cases is less than 1%, whereas for families in which the patient presents with defect in imprinting, the risk can be as high as 50%.^5^ ^,^ ^6^ Therefore, the identification of the genetic mechanism involved in each patient is a requirement for the genetic counseling and depends on the efficiency and reliability of the genetic test.

An efficient strategy for the routine diagnosis of PWS patients consists of methylation analysis, which allows the diagnosis of 99% of PWS patients, and does not require parental samples. In patients with a normal methylation pattern and normal chromosomes, a clinical reassessment is recommended to determine whether additional DNA investigations are indicated.

PWS is the main genetic cause of obesity in children, and the early approach can improve quality of care, which may give these individuals a better perspective of life.

## NATURAL HISTORY AND MANIFESTATIONS

The weight, length and body mass index of infants with PWS is often in the normal range at birth, however, they evolve with hypotonia associated with suction and feeding impairments, besides growth impairment.

According to Miller, there are seven different nutritional phases to which individuals with PWS typically progress. At the pre-natal phase (phase 0), there are reduced fetal movements and growth restriction. From birth up to 9 months of age (phase 1a) the infant is hypotonic, has feeding impairment and reduced appetite. After this period, PWS patients start gaining weight progressively. In spite of a normal caloric intake, they gain weight due to reduced metabolism (phase 1b: 9 months-2 years/phase 2a:2-4.5 years). After 4.5 years (phase 2b), the weight increase is associated with increased interest in food, but not hyperphagia. Around the age of 8 years, hyperphagia is established, characterized by food seeking and lack of satiety. Some of the individuals with PWS progress to phase 4 in adult life, when there is some leve of satiety.^7^

During early childhood, there is delayed motor and language development, with milestones achieved at about double the normal age. Intellectual and/or learning disabilities are variable and generally evident by the time the child reaches school age.^8^

## ENDOCRINOPATHIES

Individuals with PWS can present with several different endocrine disorders, most of them caused by hypothalamic-pituitary insufficiency.

### rhGH deficiency

The insulin-like growth factor 1 (IGF1) and the insulin-like growth factor binding protein 3 (IGFBP3) are low in PWS, and GH deficiency occurs in 40-100% of children.^9^

Growth hormone is an anabolic agent that increases lean body mass (LBM) and decreases fat mass in a range of conditions. It is recommended that rhGH therapy be discussed at the time of diagnosis and GH testing is not mandatory before the beginning of GH treatment.^7^ One of the most important reasons for treating PWS children with rhGH is to improve their body composition, increasing LBM and reducing fat mass, once the natural history of PWS shows increasing progressive fat mass with age.^10^ ^,^ ^11^

The rhGH therapy in combination with a healthy lifestyle makes it possible to counteract the clinical course of PWS, preventing obesity, a major threat to these patients.

Another important gain from the use of rhGH therapy is the positive effect on markers of development, such as verbal IQs, adaptive communication, cognitive skills and language.^12^ ^,^ ^13^ Children who began treatment before 12 months of age had higher nonverbal and higher composite IQs than children who began treatment between 1 and 5 years of age.^14^

The use of rhGH has not demonstrated any adverse effects on glucose parameters, lipid profile, bone mineral density or blood pressure, although insulin levels, HOMA-IR and IGF-1 may increase, as expected, during therapy. There was not a clear correlation between rhGH therapy and the development of scoliosis, but a close follow-up is recommended for its high prevalence in PWS, especially during puberty.^11^ ^,^ ^12^

Growth hormone replacement in children with PWS has well-defined benefits and risks, although data are limited to adults with PWS.^15^ The reports on rhGH treatment in adults with PWS indicate improved body composition, with increased LBM, reduced total body fat, subcutaneous adiposity and visceral fat, but with minor increase in fasting glucose after 12 months of treatment.^16^

Exclusion criteria for starting rhGH therapy in patients with PWS include severe obesity, uncontrolled diabetes, untreated severe obstructive sleep apnea, active cancer, and active psychosis. Infants and children with PWS should start on a daily dose of 0.5 mg/m^2^/day, with subsequent adjustments toward 1.0 mg/m^2^/day every 3-6 months, according to clinical response. Adults with PWS should receive a starting dose of 0.1-0.2 mg/day. Subsequent dose titration should be based on clinical response, age, and sex-appropriate IGF-I levels in the 0 to +2 SDS range.^17^

### Central Adrenal Insufficiency

In a study with 25 randomly selected PWS children performed by van Wijingaarden et al., 60% of the children presented with central adrenal insufficiency, although they had normal morning salivary cortisol levels. It suggests that adrenal insufficiency becomes apparent only during stressful conditions. Therefore, it is recommended to monitor cortisol levels before starting the rhGH treatment and assess them in situations of severe comorbidity. Van Wijingaarden et al. also suggested that hydrocortisone should be used during the acute phase of severe diseases that affect the patient (Table 1)^18^.

**Table 1:** Health Maintenance Timeline for Children with Prader-Willi Syndrome.

Lab Follow-up/Intervention	Timing	Recommendations
IGF-1 IGFBP3	At 3 months and after starting rhGH every 4-6 months	Maintain levels between +1 and +2 SD of IGF-1 during GH use
TSH Free T4	Every 12 months	With abnormal levels, conduct stricter follow-up
Glucose profile (glucose, Insulin)	Every 4-6 months	Consider metformin in females. Consider Hb1Ac and OGTT if insulin resistance
ACTH and cortisol levels	Before starting rhGH therapy and in other situations of severe comorbidity	All patients are advised to use corticosteroids for stressful conditions (during surgery or systemic disease):
		Attack with 50 mg/m^2^ of hydrocortisone
		Maintenance: 100 mg/m^2^ /day of hydrocortisone
Cholesterol and triglycerides	Every 6-12 months	Depending on weight, levels and diet
Polysomnography	Every 6-12 months	Also, with abnormal weight gain, snoring, appearance of daytime symptoms or poor school performance
Orthopedic Evaluation	Once a year	Check for scoliosis
Dental Check-Up	Every 6 months	Start teaching tooth care at a young age. Teeth should be washed twice a day, beginning as soon as the first tooth appear.

### Hypothyroidism

Hypothyroidism, particularly of central origin, is present in PWS. It has been documented in up to 25% of the individuals with PWS.

In general, thyroid function is normal at birth and treatment should not be routinely prescribed to young people with PWS. It is important to perform thyroid function screening regularly, since hypothyroidism can contribute with delayed psychomotor development, impaired linear growth during childhood and increasing fat mass and hypotonia.^19^

### Hypogonadism 

Hypogonadism is highly frequent among PWS patients, and its presentation is extremely variable. It is usually seen as genital hypoplasia, delayed puberty, incomplete pubertal development and infertility, but early puberty and premature pubarche may occur as well.

The etiology of hypogonadism in PWS is heterogeneous and is due to a combination of hypothalamic and gonadal primary dysfunction, besides the fact that sexual hormones (testosterone and estradiol) are usually below normal range.

Even though the benefits of sex hormone replacement in hypogonadism are well known, there are few reports in the literature and there is still no consensus about when or how it should be done.^20^

### Obesity

Obesity and its complications are the major causes of morbidity and mortality in individuals with PWS. There is some evidence to suggest a hypothalamic basis and an abnormal neural response to food intake.^21^ In PWS, the response os satiety is delayed, reduced or absent to food.^22^ The etiology of obesity is mostly attributed to a low basal metabolism and an elevated circulating ghrelin level than to the increased caloric intake.^23^

The maximal weight in PWS patients frequently occurs in late adolescence and early adulthood. For these patients, uncertainty and opportunity related to food consumption are a constant source of stress. Thus, they show signs of anxiety and other stress symptoms when they are responsible for their own food regulation. The main implication of these findings for the management of PWS is that the supervision of the food environment is critically important. Early counseling, caloric restriction and dietary recommendations can help keeping normal weight-for-height ratio.^7^

Up to now, pharmacologic intervention with appetite suppressants (sibutramine), anti-absorption agents (orlistat), topiramate or glucagon-like peptide 1 (GLP-1) receptor agonist have been ineffective in patients with PWS.^24^

A small series of cases reported short-term success with bariatric surgery in PWS. The results suggest there is little justification for subjecting PWS patients to the potential risks of surgical interventions, besides the risk of gastric rupture. The best recommendation for weight control in cases of severe obesity includes the use of supervised energy reduction diets with vitamin/mineral supplementation, restricted access to food, and a daily exercise regimen.^25^

### Impaired glucose tolerance and diabetes mellitus

Up to 25% of adults with PWS presented type 2 diabetes.^26^ In a multicenter study based on 274 PWS patients, altered glucose was detected in 24.4% of the patients and was significantly correlated to age, body mass index (BMI) and HOMA-IR. This study shows the strong correlation between development of altered glucose metabolism, obesity and aging.^27^

## BEHAVIORAL ISSUES AND COGNITION

Individuals with PWS present with impaired cognitive, communication and social skills. They may show a compulsive, manipulative and controlling behavior including stubbornness, episodes of temper tantrums and difficulties in changing routines. They also present a mild intellectual disability, with a mean IQ of approximately 65-70.^28^

A study comparing magnetic resonance imaging brain scan and cognitive skills showed reduction in cortical gyrus, mainly in the frontal, temporal and parietal lobes, irrespective of the genetic subtype. There was strong correlation between the alterations of these brain regions and the verbal impairment and lower IQ score in the PWS group. Frontal-parietal cortical areas are implicated in cognitive function, such as memory, attention regulation and language.^29^

PWS carriers are prone to more severe behavior problems, and as many as 85% show clinically elevated scores on standardized behavioral evaluation questionnaires. They present with high rates of obsessive-compulsive symptoms, which may express as excessive hoarding, repetitive rituals, talking too much and skin picking. Of these, skin picking seems to be the most prevalent and starts in early childhood.^30^ Aggressive behavior is common and temper tantrums occurred in 88% of the individuals aged between 4-16 years old, but the severity of these problems range widely.^31^

Studies suggest a strong association between PWS and atypical psychosis. These episodes were generally of sudden onset and often included depressive features with good response to pharmacotherapy and hospitalization.^32^

Repetitive and ritualistic behavior found in PWS individuals may differ from that seen in Obsessive-Compulsive Disorder, but may have similarities with the Autism Spectrum Disorder (ASD) characteristics.^33^ ^,^ ^34^ The denomination ‘autistic-like symptomatology’ may be appropriate when referring to PWS compulsive behavior and lack of social skills.^33^

The use of intranasal oxytocin for controlling PWS behavior has come as a possibility of treatment for these patients, but the benefits and risks are still unkown.^35^

## OTHER MANIFESTATIONS

### Sleep Disorders

Sleep disoders are frequently associated with PWS. Excessive daytime sleepiness (EDS) is the most common condition, followed by obstructive sleep apnea and narcolepsy-like symptoms. Obesity and hypothalamic dysfunction can, together, be responsible for the primary abnormalities of ventilation during sleep and determine the symptoms of EDS.^36^

In PWS individuals, unlike the general population, the EDS is not fully explained by the obstructive sleep apnea syndrome.

EDS and REM sleep disorders together are described in narcolepsy syndrome. In PWS, these narcoleptic symptoms are mainly owed to hypothalamic dysfunction, once the genetic traces of classic narcolepsy syndrome are not found in PWS.^37^

Full night polysomnography studies are recommended in children with abnormal weight gain, snoring, and appearance of daytime symptoms or poor school performance.^36^ The follow-up of sleep issues on PWS patients can be performed by means of well-established sleep-related questionnaires, in order to look for specific sleep disorders.

EDS can be treated with central nervous system stimulants, such as methylphenidate or modafinil, after complete clinical evaluation. Obstructive sleep apnea should be treated with oral appliances, continuous or bi-level positive airway pressure devices (CPAP or BiPAP) or tonsil surgery.

### Scoliosis

Scoliosis is frequently observed in children with PWS, with prevalence between 30 and 70%. Such high frequency may be partly explained by hypotonia and obesity.^38^ There are two peak ages for scoliosis in children with PWS. The presence of scoliosis under the age of 4 is most likely related to hypotonia, and the second peak, around 10 years of age, is typically idiopathic.

The earlier the change in the spinal curve is detected, the better the possibilities of treatment with casting or surgical treatment. This is indicated in severe early-onset scoliosis-kyphosis and in adolescents near skeletal maturity. Surgical complications are the risk of paraplegia (20%) and other major problems (30% of deep infections, pneumonia, hook out).^39^

### Gastrointestinal Disorders

There are only few studies investigating the gastrointestinal function of individuals with PWS. Some studies have found either normal or slow gastric emptying in PWS. Serious events with gastric rupture and necrosis followed by death have been reported and may be attributed to slow gastric emptying and absence of vomit.^40^ ^,^ ^41^

The incidence of constipation is around 40% in adult PWS patients, which is considerably higher than reported in the general population. They also have an increased prevalence of palpable stool in the rectum and prolonged gastrointestinal transit time.^42^

### Coagulation Disorders

Although thromboembolic events (TEs) have been recently reported in patients with PWS, there is no specific guideline for prophylaxis of TEs in this population of patients. We recommend that inpatients should be evaluated for risk of TE and receive mechanical prophylaxis (early end frequent ambulation, compression stockings, intermittent pneumatic compression devices and venous foot pumps), because they counteract most of the components of the Virchow’s triad and are not associated with any risk of bleeding. Combined mechanical and pharmacological prophylaxis should be considered only in PWS patients with high risk of TEs.

### Dental issues 

Dental problems are very common in PWS. PWS individuals have shown reduction in salivary flow rate and increase in the amounts of salivary ions and proteins. They present with thick saliva which sticks to the teeth and harbors bacteria that cause tooth decay and periodontal disease.^43^

## RECOMMENDATIONS

**Figure 1:**
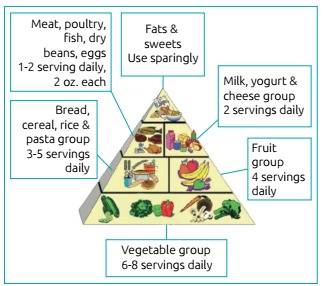
The Prader-Willi eating pyramid is divided into 5 groups: in the base, there is the vegetable group (6-8 portions a day). Above the vegetables there is the group of bread, cereal, rice and pasta group (3-5 portions a day), and next to it is the fruit group (4 portions a day). The milk, yogurt and cheese group (2 portions a day) stands right next to meat, poultry, fish, dry beans and egg group (1-2 portions a day). On the top of the pyramid there are fat and sweets, which should be eaten only sporadically (*reproduction of the figure was authorized by www.pwsausa.org*).^45^

The treatment provided in the Department of Pediatric Endocrinology of the Children’s Institute of Universidade de São Paulo for these patients is based on a follow-up protocol with four main pillars: diet (Figure 1), exercise, rhGH use and behavioral and cognitive issues, highlighted on Table 2 and on a health maintenance timeline for children with PWS (Table 1)

**Table 2:** Treatment pillars of children with Prader-Willi Syndrome.

Diet Plan	GH Therapy	Exercise Plan	Behavioral and Cognitive Strategies
Start with a 900 calories/day diet; some patients may need only 200-650 calories	Start at 3 months old	1-2 hour of daily exercise	Make sure they understand what is said: expectations and rules.
Restrict portions, according to the Prader-Willi eating pyramid (see figure 1)	It is not necessary to take the GH stimulation test or any other exam before the age of 4 (if there are no respiratory issues. After this age, a polysomnography is required)	Focus on Aerobic, Resistance and Strength	Use verbal and other cues (timers, schedules with photographs)
Ketogenic diet could be an option in patients who don’t lose weight		Working core muscles	Literally can foster misunderstandings and resentment.
Anxiety and compulsion control			Be realistic in terms of expectations of homework and class work
“No doubt, no hope, no disappointment” (use of timetables for meals)	Initial Dose of 0.5 mg/m^2^/day and maintenance of 1 mg/m^2^/day	Improving hypotonia	Use n-acetilcistein for skin picking (200-1200 mg/day)
			Be direct/ Be concrete
	Adulthood: Dose 0.5 mg/day		Compulsiveness/aggressiveness: may need a psychiatry evaluation

## CONCLUSION

The Prader-Willi Syndrome is rare genetic disease, even though it is the main genetic cause of obesity in children. Early diagnosis can prevent complications and improve quality of care throughout life. The treatment focuses on four main pillars: diet, exercise, rhGH therapy and behavioral strategies. 

If the general pediatrician becomes more familiar with PWS, the diagnosis will have a higher probability to take place and, so, treatment will start earlier. This may improve the quality of life and care for these individuals.
